# Stress Granule Assembly Can Facilitate but Is Not Required for TDP-43 Cytoplasmic Aggregation

**DOI:** 10.3390/biom10101367

**Published:** 2020-09-25

**Authors:** Nikita Fernandes, Luke Nero, Shawn M. Lyons, Pavel Ivanov, Telsa M. Mittelmeier, Timothy A. Bolger, J. Ross Buchan

**Affiliations:** 1 Department of Molecular and Cellular Biology, University of Arizona, Tucson, AZ 85721, USA; nikferns7@email.arizona.edu (N.F.); lnero@email.arizona.edu (L.N.); telsa@arizona.edu (T.M.M.); tbolger@email.arizona.edu (T.A.B.); 2 Department of Medicine, Harvard Medical School, Boston, MA 02115, USA; smlyons1@bu.edu (S.M.L.); pivanov@rics.bwh.harvard.edu (P.I.); 3 Division of Rheumatology, Immunity and Inflammation, Brigham and Women’s Hospital, Boston, MA 02115, USA

**Keywords:** TDP-43, stress granules, P-bodies

## Abstract

Stress granules (SGs) are hypothesized to facilitate TAR DNA-binding protein 43 (TDP-43) cytoplasmic mislocalization and aggregation, which may underly amyotrophic lateral sclerosis pathology. However, much data for this hypothesis is indirect. Additionally, whether P-bodies (PBs; related mRNA-protein granules) affect TDP-43 phenotypes is unclear. Here, we determine that induction of TDP-43 expression in yeast results in the accumulation of SG-like foci that in >90% of cases become the sites where TDP-43 cytoplasmic foci first appear. Later, TDP-43 foci associate less with SGs and more with PBs, though independent TDP-43 foci also accumulate. However, depleting or over-expressing yeast SG and PB proteins reveals no consistent trend between SG or PB assembly and TDP-43 foci formation, toxicity or protein abundance. In human cells, immunostaining endogenous TDP-43 with different TDP-43 antibodies reveals distinct localization and aggregation behaviors. Following acute arsenite stress, all phospho-TDP-43 foci colocalize with SGs. Finally, formation of TDP-43 cytoplasmic foci following low-dose chronic arsenite stress is impaired, but not completely blocked, in *G3BP1/2ΔΔ* cells. Collectively, our data suggest that SG and PB assembly may facilitate TDP-43 cytoplasmic localization and aggregation but are likely not essential for these events.

## 1. Introduction

Amyotrophic lateral sclerosis (ALS) or Lou Gehrig’s disease is a progressive neurodegenerative disease characterized by the death of upper and lower motor neurons, ultimately resulting in death, typically around 2–5 years after diagnosis. Approximately 90% and 10% of cases are sporadic and familial, respectively [1]. While the mutations associated with both the sporadic and familial forms of the disease can be varied, aggregates of hyperphosphorylated, ubiquitinated and truncated TAR DNA-binding protein 43 (TDP-43) within patients’ motor neurons and glial cells is a common feature in 97% of ALS cases [1,2,3].

TDP-43 is a nuclear RNA-binding protein that regulates a multitude of steps in the life of mRNAs from transcription to mRNA stability, as well as impacting miRNA biogenesis [4]. Numerous studies indicate that TDP-43 aggregates may reflect both a loss and toxic gain of function, although the relative contributions of both to ALS disease remain contentious [5]. Nonetheless, various studies that have targeted clearance of cytoplasmic TDP-43 have resulted in a mitigation of TDP-43 aggregation and toxicity [6,7], suggesting that prevention of TDP-43 mislocalization and aggregation in the cytoplasm may be a promising avenue of therapeutic research.

Stress granules (SGs), which are stress-induced assemblies of non-translating mRNA-protein complexes (mRNPs) [8,9,10], have repeatedly been implicated in the cytoplasmic mislocalization and aggregation of TDP-43 [11,12,13]. Supporting this, TDP-43 can localize to SGs under select stress conditions, though findings between labs are not always consistent (reviewed in [11]), possibly reflecting differences in cell lines or antibodies used. Additionally, many mutations associated with ALS are found in other SG-resident proteins (e.g., hnRNPA1, TIA-1, Matrin-3, Ataxin-2) and have in many cases been implicated in facilitating aberrant SG assembly [14,15,16,17]. Other ALS-associated mutations are linked to impaired SG clearance [18,19]. In either case, the increased persistence of SGs has been hypothesized to increase the likelihood of SG-localized TDP-43 forming aggregates that may lead to cytoplasmic mislocalization and a toxic gain of function. Finally, pathogenic TDP-43 aggregates in post-mortem ALS tissue sometimes harbor other SG components [20,21,22,23].

However, recent studies have suggested that the formation of cytoplasmic TDP-43 aggregates can occur either as an indirect consequence of SG assembly or be completely independent of SG assembly. For example, sequestration in SGs of HDAC6, which promotes autophagic turnover of protein aggregates [24], has been proposed to promote accumulation and aggregation of transfected mutant forms of TDP-43 prone to aggregation or lacking a nuclear localization signal (NLS); such forms of TDP-43 themselves, however, show limited SG colocalization [20]. Moreover, endocytic uptake of amyloid-like WT TDP-43 fibrils in neuroblastoma cells, or increased levels of cytoplasmic-localized TDP-43 in neuroblastoma or iPSC-derived motor neuron cells, can provoke the formation of TDP-43 liquid-like foci that only in rare instances harbor classical SG marker proteins. Such TDP-43 foci convert to a less-dynamic aggregate state following arsenite stress [25]. Additionally, TDP-43 amyloid-like “myo-granules” (cytoplasmic TPD-43 aggregates), which are likely nucleated by sarcomeric mRNAs, are observed during muscle cell differentiation that also lack canonical SG marker proteins [26]. Finally, recruitment of TDP-43 to SGs has even been suggested to maintain TDP-43 in a dynamic non-aggregated state, with SG-distinct non-dynamic TDP-43 aggregates also forming following arsenite or heat shock stress in HEK293 cells [27]. These works suggest that cytoplasmic TDP-43 assemblies may arise independently of SGs; what remains unclear, however, is whether SGs play any facilitating role, whether this varies in a cell context-dependent manner or whether SGs are in fact completely dispensable for cytoplasmic TDP-43 formation.

In this work, we utilized both yeast and human cell line models impaired in assembly of SGs, and/or related mRNP foci, termed P-bodies (PBs), to directly test the role of these mRNP granules in regulating TDP-43. We found that formation of cytoplasmic TDP-43 foci in yeast and human cell models shows a clear initial association with SGs, and at least in yeast later exhibits lower SG colocalization but increased localization with PBs. However, our yeast studies indicate no consistent relationship between the ability to form SGs or PBs and the aggregation, toxicity or abundance of TDP-43 protein. TDP-43 toxicity also does not strictly correlate with TDP-43 abundance or aggregation. In human cells, endogenous TDP-43 exhibits distinct localization phenotypes depending on the antibody utilized, which has implications for prior works in the field. Finally, cells deficient in SG assembly show impaired assembly of TDP-43 cytoplasmic foci during and following chronic stress. Together, our work is consistent with the notion that the ability to form SGs can facilitate TDP-43 cytoplasmic localization and aggregation under some circumstances but are not essential to this process.

## 2. Materials and Methods

### 2.1. Yeast Strains and Growth Conditions

The strains used in this study are described in Supplementary Table S1. The strains knocked out for specific genes were obtained from the Yeast Knockout Collection. Strains were grown on YPD (yeast extract peptone dextrose) or synthetic media (VWR Glucose 2%, Difco Yeast Nitrogen Base 0.17%, Fisher Ammonium sulfate 5 g/L, appropriate amino acids and nucleotides). All strains were grown at 30 °C in shaking water baths. A standard lithium acetate technique was used for yeast transformations.

### 2.2. Plasmids

All plasmids utilized in this study are listed in Supplementary Table S1. A previously described tiling array collection [28] featuring yeast genes on multi-copy plasmids in their native genomic context was used for over-expression analyses.

### 2.3. Yeast Serial Dilution Assays

To induce TDP-43 expression, strains with plasmids expressing *GAL1*-driven TDP-43 or empty vector plasmids were cultured overnight in 0.25% galactose and 1.75% sucrose. The overnight cultures were back diluted to 0.1 OD_600nm_ in identical media, followed by growth at 30 °C to mid-log (~0.5 OD_600nm_), after which the cultures were adjusted to 0.2 OD_600nm_, serially diluted (1/5 dilutions), and spotted onto agar media containing either 2% glucose, 0.25% galactose/1.75% sucrose or 2% galactose. Higher levels of galactose induce stronger TDP-43 expression and thus toxicity [29]. For the yeast over-expression experiments, the appropriate over-expression plasmid/empty vector plasmid was transformed into WT BY4741 strain along with the *GAL1*-driven TDP-43 plasmid/empty vector plasmid.

### 2.4. Microscopy

**Figure 1:**
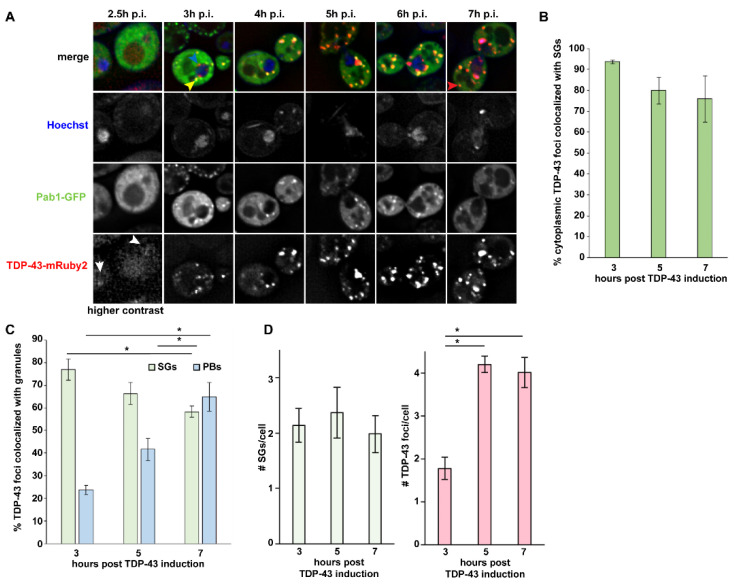
TAR DNA-binding protein 43 (TDP-43) foci predominantly initiate with stress granules (SGs). (**A**) Log-phase *Saccharomyces cerevisiae* BY4741 (WT) were transformed with pRB194-expressing TDP-43-mRuby2 and pRB015 expressing Pab1-GFP (SG marker). Hoescht was used to visualize the nucleus. TDP-43 expression induced by 2% Galactose, with timepoints post induction (p.i.) microscopically imaged as indicated. Arrowheads indicate phenotypes mentioned in the text. (**B**) % colocalization of cytoplasmic TDP-43 foci with Pab1-GFP (perinuclear-associated foci excluded). Data generated from 3 biological replicates with mean ± s.d shown. (**C**) % colocalization of all TDP-43 foci with Pab1-GFP (including peri-nuclear TDP-43 foci) or Dcp2-GFP (see Figure S1). Data generated from 3 biological replicates with mean ± s.d shown. An ANOVA with Tukey’s post-hoc test was used to assess significance (* indicates significance). (**D**) Total SG and TDP-43 cytoplasmic foci number; replicates and error bars as in (**C**). An ANOVA with Tukey’s post-hoc test was used to assess significance.

**Figure 2:**
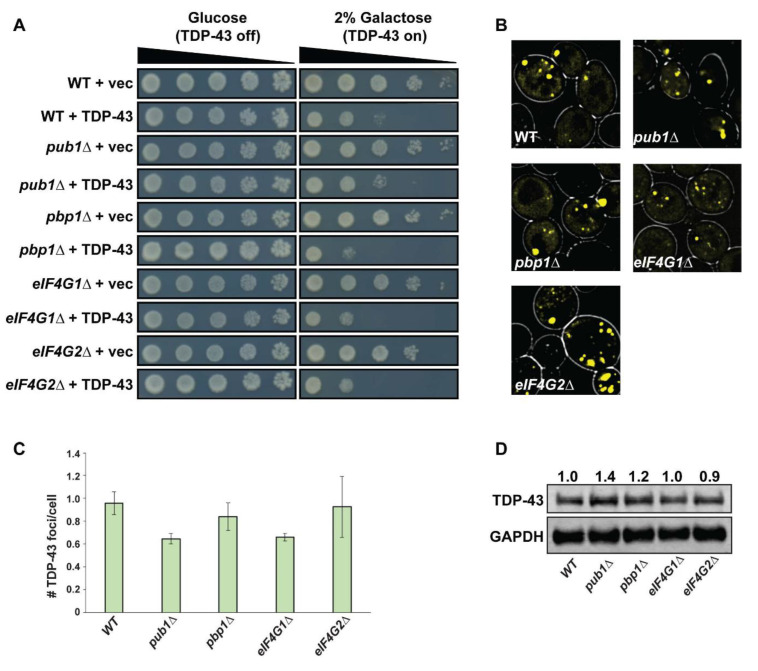
SG assembly mutants do not strikingly affect TDP-43 toxicity, foci formation or abundance. (**A**) BY4741 (WT) and indicated strains were transformed with pRB109 expressing TDP-43-YFP and examined for TDP-43 toxicity by serial dilution spotting assay. (**B**) The strains mentioned above were examined for the presence of TDP-43 foci. (**C**) Quantification of (**B**); number of TDP-43-YFP foci/cell. Data generated from 3 biological replicates with mean ± s.d shown. (**D**) Western analysis of above strains to assess steady-state TDP-43-YFP protein levels; quantification, based on two biological replicates, is normalized to GAPDH loading control.

To induce TDP-43 expression for Figure 1, strains with plasmids expressing *GAL1*-driven TDP-43 and SG/PB marker were cultured overnight in 2% sucrose. The overnight cultures were back-diluted to 0.1 OD_600nm_ in identical media, followed by growth at 30 °C to mid-log (~0.5 OD_600nm_), after which the media was switched to 2% galactose and live-cell microscopy was performed at indicated timepoints. Hoescht was added at a final concentration of 12 μg/mL to cultures 10 min prior to visualization to stain DNA. To induce TDP-43 expression (for Figure 2 onwards), strains with plasmids expressing *GAL1*-driven TDP-43 were cultured overnight in 0.25% galactose and 1.75% sucrose. The overnight cultures were back-diluted to 0.1 OD_600nm_ in identical media, followed by growth at 30 °C to mid-log (~0.5 OD_600nm_), after which live-cell microscopy was performed. For Figure S4, overnight cultures were grown in 2% sucrose. Analyses of TDP-43 aggregation similar to a method previously described for PB assembly [30] were conducted, using a Deltavision Elite (Cytiva, Marlborough, MA, USA) (100× objective) followed by image analysis using Fiji software [31]. For every yeast strain, a minimum of 100 cells/per replicate were quantified, with a minimum of 3 biological replicates examined for each microscopy dataset. For mammalian immunofluorescence experiments (see below), a minimum of 2 biological replicates were examined with 50 cells/replicate quantified.

### 2.5. Western Blots

Western blotting was carried out by standard protocols. Protein extracts were loaded onto SDS-polyacrylamide gels. Unless otherwise indicated, TDP-43 was detected by using Rabbit polyclonal anti-TDP-43 (Proteintech, Rosemont, IL, USA, 10782-2-AP) at a 1:10,000 dilution. GAPDH (Glyceraldehyde 3-phosphate dehydrogenase) was detected by using Mouse anti-GAPDH (Thermo Fisher Scientific, Waltham, MA, USA) at a 1:25,000 dilution. YFP was detected by using Rabbit anti-GFP (Abcam, Cambridge, MA, USA) at a 1:10,000 dilution. Detection was carried out by using Li-Cor fluorescently labeled secondary antibodies and the LiCor Odyssey Imaging system (LiCor, Lincoln, NE, USA). Secondary antibodies used were IRDye 800CW Goat anti-Rabbit IgG (H + L) and IRDye 800CW Goat anti-Mouse IgG (H + L) (LiCor, Lincoln, NE, USA) to detect the primary antibodies mentioned above as appropriate.

### 2.6. CRISPR/Cas9-Mediated Knockout of TDP-43

Oligonucleotides targeting TDP-43 were ordered from Integrated DNA Technologies (Integrated DNA Technologies, Coralville, IA, USA). Oligonucleotides were annealed and cloned into pCas-Guide (Origene, Rockville, MD, USA) according to manufacturer’s protocol. gRNA targeted the following sequence: GTTTGTGGGGCGCTGTACAG. Cloned pCas-guide was co-transfected with pDonor-D09 (GeneCopoeia, Rockville, MD, USA) using Lipofectamine 2000 (Invitrogen by Thermo Fisher Scientific, Waltham, MA, USA) into U-2 OS cells. The following day, cells were treated with puromycin (1.5 μg/mL). After 48 h of selection, puromycin was removed. Single-cell clones were generated by limiting dilution.

### 2.7. U-2 OS Cell Culture and Immunohistochemistry

U-2 OS cells were grown in 8-well chamber slides (Ibidi, Fitchburg, WI, USA, 80821), fixed with 4% paraformaldehyde for 15 min, permeabilized with 0.1% Trion X-100 for 10 min and then blocked in 5% goat serum for 1 h. Cells were incubated with indicated primary antibodies at RT for 2 h. Cells were washed three times and then incubated with Alexa Fluor conjugated secondary antibody (Thermo Fisher Scientific, Waltham, MA, USA, 1:1000 dilution) for 1 h at room temperature. After three washes with PBS, cells were stained with DAPI (Thermo Fisher Scientific, Waltham, MA, USA, P36931) and imaged using a Deltavision Elite microscope (Cytiva, Marlborough, MA, USA) with a 20× objective. Antibodies used for immunofluorescence were α-TDP-43 (Proteintech, Rosemont, IL, USA, 60019-2-Ig dilution 1:200, Proteintech, Rosemont, IL, USA, 10782-2-AP dilution 1:200), α-phospho(S409/S410)-TDP43 (Proteintech, Rosemont, IL, USA, 22309-1-AP dilution 1:200), α-G3BP (Abcam, Cambridge, MA, USA, ab181149 dilution 1:500), α-TIAR (Santa Cruz, Dallas, TX, USA, sc-398373 dilution 1:200). *G3BP1/2ΔΔ* cell lines were generated as previously described [32].

## 3. Results

### 3.1. TDP-43 Foci Formation Largely Initiates at SGs in Yeast, Followed by Later PB Localization

To assess the importance of SGs and PBs on initial TDP-43 aggregation events, we induced expression of TDP-43 in yeast and examined how TDP-43 foci formation over time relates to both structures. TDP-43 expression in yeast recapitulates aggregation and toxicity phenotypes associated with human cell models [33] and allows assessment of the effect of various SG and PB assembly mutants (see later). Additionally, yeast has a long precedent in revealing physiologically relevant insight into the study of aggregation-prone proteins associated with neurodegenerative diseases [34]. We used a TDP-43-mRuby2 plasmid, under control of a Galactose-inducible *GAL1* regulatable promoter, which behaves similarly to a previously described yeast TDP-43-YFP model [33,35] in terms of foci formation [29]. Pab1-GFP and Dcp2-GFP single-copy plasmids, under native promoters, were used to visualize SGs and PBs respectively, with Hoescht added to visualize DNA. We observed the following trends regarding TDP-43 foci formation, which may indicate or be a precursor to TDP-43 aggregation.

At 2.5 h of induction, TDP-43-mRuby2 signal was not readily discernible above vacuolar auto-fluorescent background levels; some faint diffuse nuclear signal (white arrowheads) was observed in a fraction of cells, consistent with prior observations of nuclear TDP-43 accumulation under low expression conditions [33]. Notably, faint SGs are induced in 38.7% of cells at 2.5 h, clearly appearing before the emergence of almost all TDP-43 foci (Figure 1A and Supplementary Table S2). At 3 h post-induction, TDP-43 foci begin appearing with two primary phenotypes; one is the association of often multiple TDP-43 foci with the nuclear membrane (Figure 1A; 10.0% of cells, blue arrowhead), reminiscent of perinuclear granules [36]. Cytoplasmic TDP-43 foci (Figure 1A; yellow arrowhead) also appear at this point (15.4% of cells) and become the much more common foci phenotype as induction time progresses. Importantly, 93.6% of cytoplasmic TDP-43 foci (not including nuclear membrane foci) first appear directly colocalized with SGs (Figure 1B). However, TDP-43-SG colocalization frequency slightly reduces over time to 75.9% at 7 h post-induction, with SG-independent TDP-43 foci increasingly emerging (Figure 1A, red arrowhead; and Figure 1C). Contrary to this trend, at 3 h post-induction, only 23.6% of TDP-43 foci colocalize with PBs; this colocalization with PBs increases over time, however, reaching 64.9% at 7 h post-induction (Figure S1 and Figure 1C).

In summary, our data suggest that early in a TDP-43 induction timecourse (2.5 h), SGs are induced, which later serve as sites of initial TDP-43 foci appearance in the majority of cases; SG-independent perinuclear TDP-43 foci are also observed at 3 h, which is distinct from previously described intranuclear TDP-43 foci [33]. Over time, TDP-43 foci transition to a more SG-independent and/or PB-associated state.

### 3.2. Yeast SG Assembly Mutants Show Minor or No Effect on TDP-43 Toxicity and Foci Formation

As most TDP-43 foci initially appear within yeast SGs (Figure 1A), and the fact that SG assembly is implicated in TDP-43 and ALS pathology [12], we next directly assessed whether the ability to form SGs alters TDP-43 toxicity, foci formation and protein abundance in yeast. We expressed *GAL1*-driven TDP-43-YFP from a single copy plasmid in previously described SG assembly mutants [37,38] and assessed toxicity by a growth assay (Figure 2A), foci formation after 24 h by microscopy (Figure 2B,C) and protein levels via Western blotting (Figure 2D). If SGs indeed facilitate TDP-43 cytoplasmic accumulation, foci formation and toxicity, a simple prediction is that SG assembly mutant yeast may show a reduction in these phenotypes relative to WT controls.

We found that TDP-43 toxicity in previously identified SG assembly mutants was generally very similar to that in WT strains; *pbp1Δ* strains slightly increased TDP-43 toxicity in our hands (contrary to prior findings [39]), whereas *pub1Δ* strains showed a very slight suppression of TDP-43 toxicity, but none of these results are striking (Figure 2A). Additionally, we see a slight but not striking growth defect in *eIF4G1Δ* and *eIF4G2Δ* strains expressing TDP-43. Thus, impairing SG assembly, to the extent that this is possible in yeast (see below), has little effect on TDP-43 toxicity.

We also observed no correlation between the previously reported ability of *pub1Δ*, *pbp1Δ*, *eIF4G1Δ* or *eIF4G2Δ* strains to form SGs and the propensity of TDP-43 to form cytoplasmic foci (Figure 2B,C). While the *pub1Δ* strain background, which is the most impaired in stress-induced SG assembly of the four strains tested [37,38], does show modestly lower levels of TDP-43-YFP foci formation (40% decrease compared to WT; not significant), an *eIF4G1Δ* strain shows a similar decrease, yet possesses the mildest of SG assembly defects of our four strains [37]. Finally, no striking difference in TDP-43 protein levels was observed in any of the SG assembly mutant strains (Figure 2D), and what changes in levels are observed are not mirrored by correlative changes in TDP-43 aggregation propensity.

A caveat of the above is that none of the mutants tested completely block yeast SG assembly under stress [37,38]; indeed no yeast strain to date has been identified that is completely deficient in SG assembly, likely reflecting a redundant assembly process. Nonetheless, our initial yeast SG mutant data suggest that impairing SG assembly does not significantly affect TDP-43 foci formation, levels or toxicity.

### 3.3. Yeast PB Mutants, with the Exception of edc3Δ pat1Δ, Show No Effect on TDP-43 Foci Formation or Toxicity

While SGs have received much attention as putative sites of TDP-43 concentration, leading to aggregation and possibly enhanced toxicity, there has been less focus on whether PBs could play a similar role. Additionally, in yeast, PB assembly can stimulate SG assembly, and mutants exist (*edc3Δ pat1Δ*, *edc3 lsm4Δc*, *dhh1Δ pat1Δ*) that exhibit stronger blocks in PB assembly relative to the above tested SG mutants [37,40]. Therefore, as before, we assessed the effect of PB assembly mutants on TDP-43 toxicity, foci formation and overall protein levels.

TDP-43 toxicity was not significantly altered in any PB assembly mutant. Likewise, TDP-43 foci formation was also not altered in any PB mutant except for *edc3Δ pat1Δ*, in which TDP-43 foci formation is severely impaired (Figure S2A,B). Finally, TDP-43 steady-state protein levels were also much lower in the *edc3Δ pat1Δ* strain (Figure S2C).

In summary, while most PB assembly mutants have a negligible effect on TDP-43 foci formation, *edc3Δ pat1Δ* yeast does exhibit an inhibitory influence. While this is likely explained by a decrease in TDP-43 levels in this strain, given our earlier time-course data (Figure 1), this could also suggest that PBs may help maintain a subpopulation of TDP-43 foci after their initial formation.

### 3.4. Over-Expression of SG and PB Assembly Proteins Causes Variable Effects on TDP-43 Toxicity, Foci Formation and Protein Abundance

We next asked whether attempting to enhance SG or PB assembly, rather than impair it, might affect TDP-43 toxicity or foci formation. To address this, we over-expressed proteins associated with SG or PB assembly [37,40,41,42] in our TDP-43-YFP yeast toxicity model. Proteins of interest were over-expressed via a multi-copy, native promoter plasmid system derived from a genomic tiling array collection [28]. While our assembly mutant data for both SGs and PBs yielded few striking effects, several mutations associated with overall increased SG assembly have been implicated in ALS pathology, hence our interest in this question. To elucidate the differing severity of effects of SG/PB protein over-expression on TDP-43 toxicity, we conducted spotting assays with a “low” (0.25% Galactose) and “high” (2% Galactose) TDP-43 expression regime.

**Figure 3:**
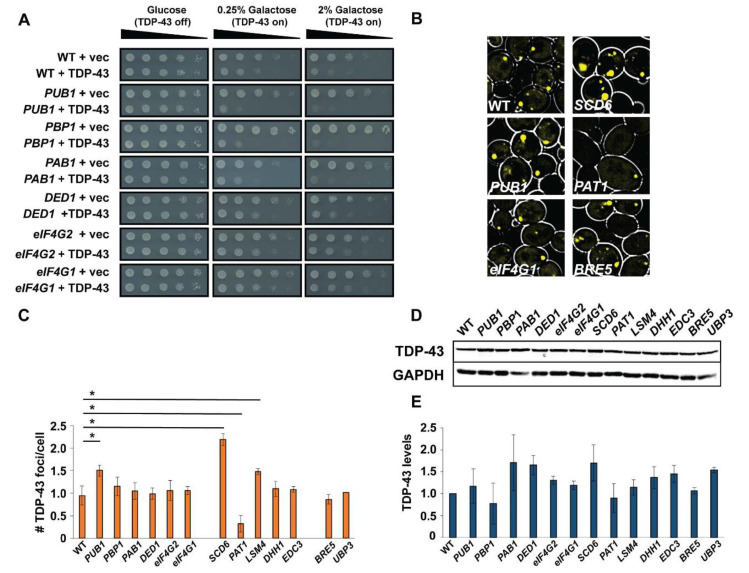
Over-expression of SG proteins has variable effects of TDP-43 toxicity, foci formation and abundance. (**A**) BY4741 (WT) and indicated SG assembly protein over-expression strains were transformed with pRB109 expressing TDP-43-YFP and multi-copy plasmids containing indicated genes of interest under native promoter control. TDP-43 toxicity was examined by serial dilution spotting assay. (**B**) Select microscopy images of TPD-43-YFP foci in cells over-expressing indicated SG/PB assembly proteins. (**C**) Quantification of average TDP-43 foci/cell for the complete SG/PB protein over-expression dataset. Data generated from 3 biological replicates with mean ± s.d shown. An ANOVA with Dunnet’s post-hoc test was used to assess significance. (**D**) Western blot of TDP-43-YFP protein levels in indicated over-expression strains. (**E**) Quantitation of data in (**D**), based on 2 biological replicates, is normalized to GAPDH loading control. An ANOVA with Dunnet’s post-hoc test was used to assess significance (* indicates significance).

Interestingly, several over-expression strains exhibited increased TDP-43 toxicity relative to WT, including *PUB1* (SG assembly protein), *PBP1* (SG assembly protein), *SCD6* (PB assembly protein), *PAT1* (PB assembly protein), *BRE5* (SG assembly protein) and *UBP3* (SG assembly protein) (Figure 3 and Figure S3). While not examined in this work, we recently observed that *bre5Δ* and *ubp3Δ* strains exhibit reduced TDP-43 toxicity and protein abundance [43]. Conversely, two over-expression strains showed suppression of TDP-43 toxicity, namely *eIF4G1* and *eIF4G2*. All other strains showed essentially no difference in TDP-43 toxicity (Figure 3A and Figure S3A,B). As for effects on TDP-43 foci formation, *PUB1*, *SCD6* and *LSM4* over-expression significantly increased TDP-43 foci (Figure 3B,C), whereas *PAT1* over-expression significantly reduced TDP-43 foci. All other strains showed no significant difference in TDP-43 foci formation. Finally, TDP-43 protein levels showed no significant differences in the strains examined (Figure 3D,E).

A simple model would be that increasing TDP-43 toxicity correlates with increased TDP-43 abundance and increased foci formation, but few of our over-expression strains exhibit this trend; only *PUB1* and *SCD6* over-expression adhere to this. In contrast, *PAT1* over-expression increases TDP-43 toxicity but strikingly reduces TDP-43 foci numbers/cell. Additionally, *DED1* over-expression increases TDP-43 levels but does not result in significant changes to TDP-43 toxicity or foci formation. Even stronger *DED1* over-expression via a *GAL1*-promoter plasmid, which does visibly increase TDP-43 foci size, still fails to exhibit significant toxicity effects (Figure S4). In summary, the toxicity of TDP-43 in yeast does not depend strictly on TDP-43 levels or aggregation propensity, nor do SG or PB-assembly or protein over-expression strains exhibit consistent trends upon TDP-43 phenotypes.

### 3.5. Endogenous TDP-43 Colocalizes with Mammalian SGs, although Antibodies Utilized Affect Detection

**Figure 4:**
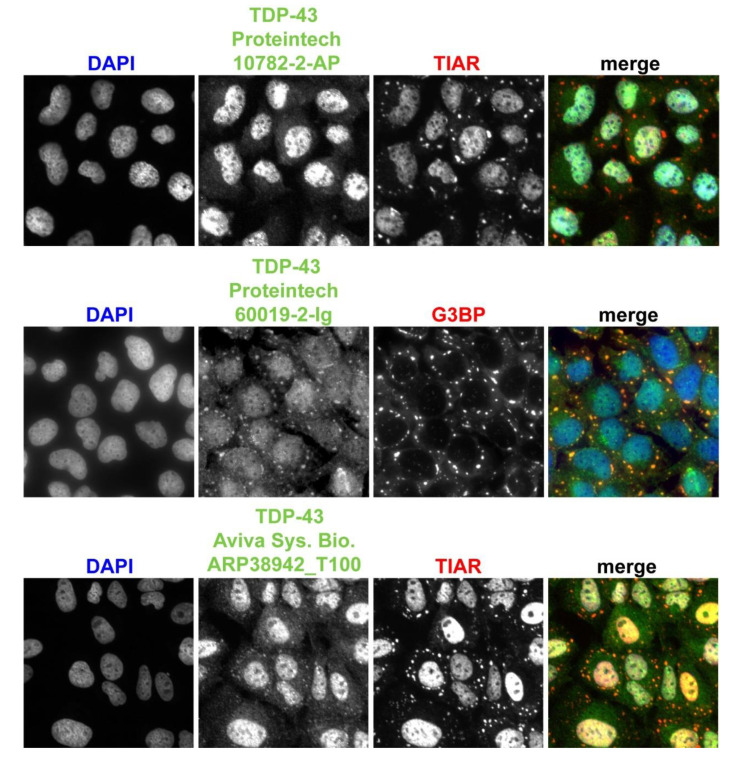
Detection of endogenous TDP-43 in arsenite-induced SGs varies in an antibody-dependent manner. U2-OS (WT) cells were stressed with 0.5 mM sodium arsenite for 1 h followed by immunofluorescence and imaging with indicated TDP-43 antibodies. SG localization was verified with either TIAR or G3BP staining as indicated.

We next chose to examine TDP-43 phenotypes in SG assembly mutants in mammalian cells, where more robust genetic blocks of microscopically visible SGs have been observed; specifically, *G3BP1/2ΔΔ* cells [32]. However, while several studies have shown that TDP-43 colocalizes to SGs, others have failed to see this colocalization [11]. Recent work suggests that these conflicting results may reflect the existence of different TDP-43 protein species, resulting from alternative splicing and protein cleavage events, which are in turn differentially recognized by various commonly used TDP-43-specific antibodies [3,44]. Thus, we first began by examining endogenous TDP-43 protein localization to SGs in WT U2-OS cells following acute arsenite stress (1 h, 0.5 mM) using a variety of TDP-43 antibodies (Figure 4).

With a commonly used polyclonal antibody [44,45] (Proteintech, Rosemont, IL, USA, 10782-2-AP), we observed only very faint localization of TDP-43 to some SGs, with the vast majority of signal remaining nuclear. In comparison, we observed clearer colocalization of TDP-43 to SGs by using a monoclonal TDP-43 antibody (Proteintech, Rosemont, IL, USA, 60019-2-Ig) and with a polyclonal antibody that specifically recognizes TDP-43′s C-terminus (Aviva Systems Biology, San Diego, CA, USA, ARP38942_T100). Interestingly, the Proteintech antibodies have been reported to recognize almost the same epitope (amino acids 203–207 for 10782-2-AP polyclonal; 203–209 for the 60019-2-Ig monoclonal) based on truncation and Western blot analysis [46]; thus, it is not clear what could account for the differences in TDP-43 localization that we observe here. In contrast, the Aviva antibody immunogen peptide encompasses amino acids 337–386.

To assist the TDP-43 community, we tested the specificity of these antibodies (Proteintech, Rosemont, IL, USA, 10782, 22309, Aviva ARP38942) via Western blotting of cells either harboring full length or an N-terminally truncated TDP-43-GFP species (and endogenous TDP-43) [29] and compared to detection with an αGFP antibody. All tested TDP-43 antibodies recognized endogenous TDP-43, TDP-43-GFP and TDP-35-GFP (amino acids 90–414; see Figure S5). Furthermore, full-length endogenous TDP-43 signal is lost as expected in *TDP-43Δ* cells (Figure S5). Finally, immunofluorescence (IF) of unstressed WT and *TDP-43Δ* cells revealed significant loss of nuclear signal with all three antibodies as expected, though the monoclonal Proteintech antibody exhibited non-specific cytoplasmic signal in the *TDP-43Δ* cell line. We also conducted identical validation analyses using a phospho-TDP-43 antibody (S409/410; Proteintech, Rosemont, IL, USA, 22309-1-AP); however, contrary to other reported observations with this antibody in distinct cell lines [20,47], we observed significant diffuse nuclear signal in both the presence and the absence of arsenite stress and SG-localizing foci, even in *TDP-43Δ* cells. In summary, endogenous TDP-43 indeed localizes to SGs, but the extent of colocalization observed depends strongly on the antibody used.

### 3.6. SG Assembly May Positively Impact Formation of Persistent TDP-43 Cytoplasmic Foci under Chronic but Not Acute Stress

Various models for how SGs may promote TDP-43 aggregation exist, predominantly based on human cell line and ALS patient tissue data. The first suggests that TDP-43 initially localizes in SGs, leading to formation of a persistent, non-dynamic TDP-43-containing SG aggregate; certain ALS histological studies and cell line studies with inducible SG assembly support this [22,48]. A second model is that SGs may merely transiently concentrate TDP-43 before disassembling (after stress adaptation or stress removal), leaving TDP-43 aggregates in their wake [49]. A third possibility is that TDP-43 aggregation proceeds completely independently of SGs and/or that TDP-43 localization in SGs may even inhibit misfolding and/or phosphorylation events associated with the former; this was recently suggested to occur during acute stress [45] (all models reviewed in [11]).

A simple test of the above models is to examine whether TDP-43 cytoplasmic aggregation is altered in cells unable to form cytoplasmic SGs. If SGs do facilitate TDP-43 aggregation, SG assembly mutants should exhibit fewer TDP-43 foci; if TDP-43 aggregation is SG-independent, no effect on TDP-43 aggregation in SG mutants would be expected. We utilized *G3BP1/2ΔΔ* cell lines, which are virtually devoid of microscopically visible SGs under arsenite stress (a stronger SG impairment than any yeast SG assembly mutants we earlier observed), and examined formation of TDP-43 cytoplasmic foci over both an acute high dose (1 h, 0.5 mM) and chronic low dose (16 h, 50 μM) arsenite stress timecourse, as well as after stress recovery.

**Figure 5:**
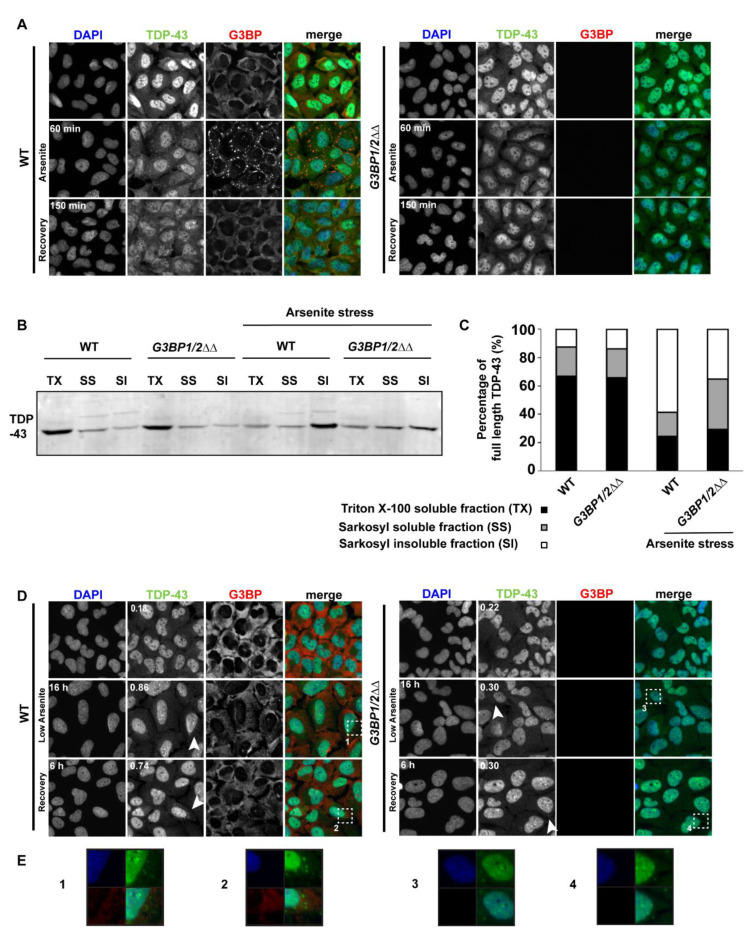
SG assembly mutants impair the formation of TDP-43 cytoplasmic foci in acute and chronic stress. (**A**) U2-OS (WT) and *G3BP1/2ΔΔ* cells were unstressed, stressed with 0.5 mM sodium arsenite for 1 h or stressed with 0.5 mM sodium arsenite for 1 h followed by recovery for 2.5 h. Immunofluorescence for TDP-43 utilized the Proteintech 60019-2-Ig antibody, with G3BP staining serving as an SG marker (and conformation of KO status). (**B**) Endogenous TDP-43 solubility in WT and *G3BP1/2ΔΔ* cells was determined via differential solubilization using Triton X-100 (TX), Sarkosyl (SS) and SDS (SI). Western blotting was conducted with the Proteintech 10782-2-AP antibody. (**C**) Quantification of (**B**). (**D**) U2-OS (WT) and *G3BP1/2ΔΔ* cells immunostained as in (**A**) were left unstressed, stressed with 0.05 mM sodium arsenite for 16 h or stressed with 0.05 mM sodium arsenite for 16 h, followed by recovery for 6 h. Numbers indicated in TDP-43 column indicate average number of TDP-43 foci/cells (see also Supplementary Table S2). Arrowheads indicate cells with TDP-43 foci. Immunofluorescence (IF) was carried out as in (**A**). (**E**) Zoom panels indicated by white boxes in (**D**).

During acute arsenite stress, only WT cells formed SGs as expected, into which TDP-43 readily colocalized; 94% colocalization at 1 h (Figure 5A). We also measured TDP-43 solubility in WT and *G3BP1/2ΔΔ* cell lines following acute arsenite stress to determine if SG assembly and TDP-43 localization there may impact TDP-43 solubility. Notably, TDP-43 solubility clearly decreased more in WT cells than in *G3BP1/2ΔΔ* cells following arsenite stress, based on Sarkosyl detergent solubility [50], though some increase in TDP-43 insolubility was still observed in *G3BP1/2ΔΔ* cells (Figure 5B,C). This indicates that SG assembly facilitates but is not absolutely required for decreases in TDP-43 solubility following acute arsenite stress. Despite this, following 2.5 h of recovery, only rarely did we observe TDP-43 foci that colocalized with SGs. In *G3BP1/2ΔΔ* cells, SGs were not observed and TDP-43 cytoplasmic foci were only very rarely observed. This suggests that SGs facilitate TDP-43 foci formation (in SGs) under acute arsenite stress conditions, but this does not result in persistence of TDP-43 foci thereafter once SGs have disassembled.

In contrast, during chronic arsenite stress, three key observations were made (Figure 5B). First, a significantly higher number of cells with cytoplasmic TDP-43 foci were visible at 16 h in WT cells versus *G3BP1/2ΔΔ* cells, (0.86 foci/cell, 40% cells with TDP-43 foci versus 0.3 foci/cell, 10% cells with TDP-43 foci, see white arrowheads). Second, following 6 h of recovery, there was a higher number of TDP-43 foci present in WT cells (0.74 foci/cell, 22% cells with TDP-43 foci) versus *G3BP1/2ΔΔ* cells (0.3 foci/cell, 14% cells with TDP-43 foci; see white arrowheads), with again only occasional overlap with G3BP foci signal in WT cells. Third, in WT cells, we rarely observed microscopically visible SG formation with this concentration of arsenite, including at earlier timepoints. TDP-43 foci colocalized with SGs in only 1.9% of cases at the 16 h chronic stress timepoint; thus, a clear relationship between SG assembly and TDP-43 foci formation under this condition remains to be determined (see discussion). In summary, *G3BP1/2ΔΔ* cells are impaired, though not completely blocked, in TDP-43 foci formation under a chronic stress regime and following 6 h of recovery; this suggests a facilitating but non-essential role for mammalian SGs in TDP-43 aggregation under chronic stress.

### 3.7. TDP-43 Protein Levels Decrease Following Stress in SG Assembly Mutant Cells

**Figure 6:**
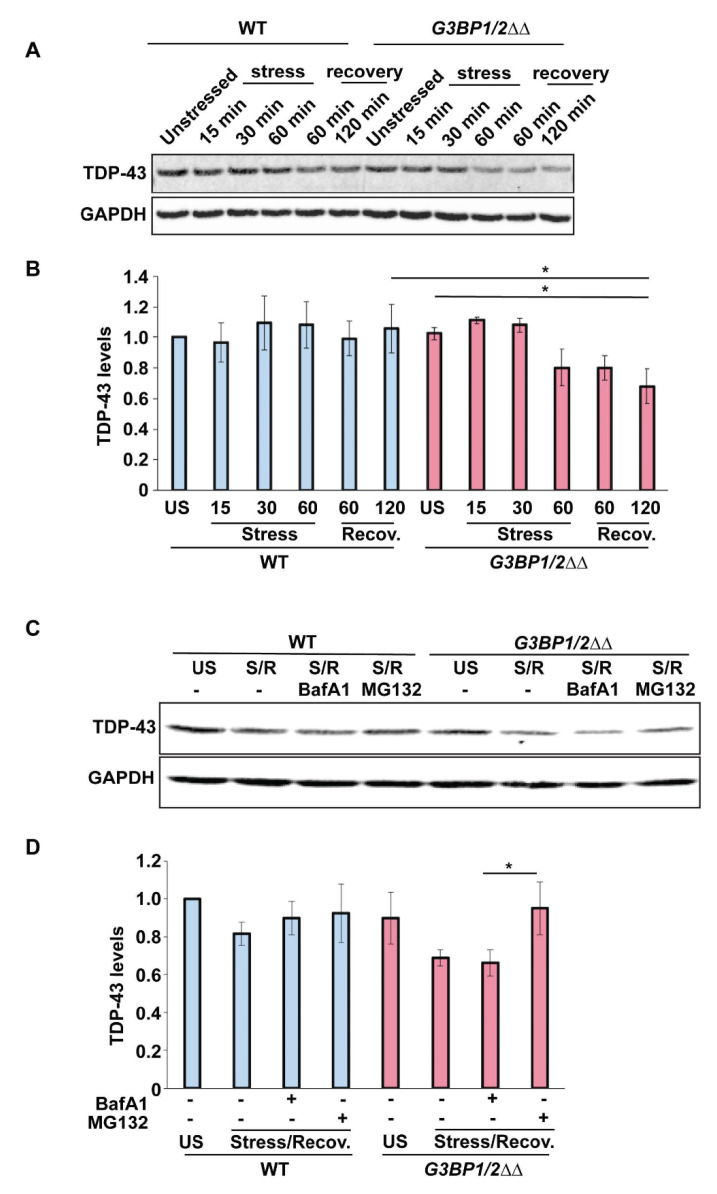
SG assembly correlates with maintenance of TDP-43 protein levels during stress recovery. (**A**) U2-OS (WT) and *G3BP1/2ΔΔ* cells were left unstressed, stressed with 0.5 mM sodium arsenite for 1 h or stressed with 0.5 mM sodium arsenite for 1 h followed by recovery for 2 h. Westerns were carried out to assess TDP-43 protein levels. (**B**) Quantitation of data in (**A**). Data generated from 3 biological replicates with mean ± s.d shown; TDP-43 levels were normalized to GAPDH loading controls. An ANOVA with Tukey’s post-hoc test was used to assess significance. (**C**) U2-OS (WT) and *G3BP1/2ΔΔ* cells were left unstressed (US), stressed with 0.5 mM sodium arsenite for 1 h, followed by recovery for 2 h (S/R). Proteasomal (10 μM MG132) and Lysosomal (0.5 μM Bafilomycin A1) inhibitors were added co-incident with arsenite addition. Westerns carried out as described in (**A**). (**D**) Quantitation of (**C**), as described in (**B**). An ANOVA with Tukey’s post-hoc test was used to assess significance (* indicates significance).

We next examined whether the ability to form SGs affects endogenous TDP-43 protein levels before, during or following recovery from an acute arsenite stress. In WT cells, no significant change in TDP-43 levels was observed over an hour of stress or after 1 or 2 h of stress recovery (Figure 6A,B). In *G3BP1/2ΔΔ* cells, we observed no difference in TDP-43 levels compared to WT prior to stress. However, we did observe a modest 20% decrease in TDP-43 protein levels by 1 h of acute arsenite stress, which decreased a further 10% by 2 h of recovery (statistically significant). This preliminary data suggests that SG assembly may help maintain TDP-43 protein levels during and after acute stress.

To understand this result further and examine the possible contribution of protein turnover mechanisms, we re-examined endogenous TDP-43 protein levels before stress and after 1 h of stress and 2 h of recovery in the presence or absence of MG132 (proteasomal inhibitor) or Bafilomycin A1 (InvivoGen, San Diego, CA, USA) (lysosomal inhibitor). While WT cells showed no significant change in TDP-43 levels under any of the examined conditions, *G3BP1/2ΔΔ* cells again revealed a decrease in TDP-43 levels following stress, which was prevented by the presence of MG132, but not Bafilomycin A1. This preliminarily suggests that in *G3BP1/2ΔΔ* cells, the reduction in TDP-43 levels during and following recovery from an acute stress is related to increased proteasomal turnover of TDP-43. We cannot however rule out that other factors (e.g., altered translation rates) may impinge on this result.

## 4. Discussion

Our data, in yeast and human cells, suggest that >90% of cytoplasmic (non-nuclear-associated) TDP-43 foci first form in association with microscopically visible SGs. In yeast, inducing TDP-43 expression alone induces SGs, which appear shortly before visible TDP-43 foci appear (Figure 1). It is unclear why TDP-43 expression induces SGs in yeast. However, a reasonable speculation based on prior mammalian work is that TDP-43′s RNA-binding activity and intrinsically disordered (IDR) C-terminus confer a high valency potential to interact with other SG components and enhance existing yeast SG assembly mechanisms. TDP-43′s RNA binding activity and IDR are at least necessary for its own foci-forming ability in yeast [33]. Regardless, there is a physical proximity between SG and TDP-43 foci at the time of the latter’s emergence, suggesting that SGs provide a favorable environment for TDP-43 aggregation. In U2-OS cells during acute arsenite stress, endogenous TDP-43 also initially forms foci within SGs (Figure 5). This finding mirrors another recent time-course study using different cell lines and an NLS-mutant form of GFP-tagged TDP-43 [25]. This study also reported formation of SG-independent foci at later timepoints (80–90 min onwards), which we are unable to assess in our experiments due to accumulating cell death beyond the 60 min timepoint; nonetheless, such data suggest that SGs may serve at least a transient role in TDP-43 foci formation.

To our knowledge, the localization of TDP-43 in PBs has not previously been addressed, certainly during a time-course analysis. We do not know why in yeast TDP-43 initially localizes in SG-like foci and over time shows increasing localization to PBs. SGs and PBs interact physically and can exchange certain mRNP components [37,51,52]. PBs may also be less dynamic than SGs given that they are present in cells under normal growth, whereas SGs are absent, and given that, for the select group of SG and PB proteins for which fluorescence recovery after photobleaching (FRAP) experiments have been conducted, generally speaking, PB proteins show lower exchange rates [8]. Thus, one speculative possibility is that while TDP-43 favors initial interactions with SG proteins, progressive sampling/exchange of TDP-43 with less dynamic PB compartments, coupled with more frequent turnover of SGs, may make enrichment of TDP-43 in PBs over time more likely.

Recently, cytoplasmic TDP-43 aggregates and SGs have been strongly linked to nucleocytoplasmic trafficking defects, which are now a pathology hallmark of interest in ALS. Specifically, forms of TDP-43 prone to aggregation or cytoplasmic accumulation interact and sequester nucleocytoplasmic machinery, leading to impairment of import and export processes and altered nuclear pore and lamina morphology [53,54]. SG assembly itself also affects nucleocytoplasmic transport via sequestration of key importins, exportins and other nuclear-pore associated factors [55], and has been proposed to act as a driver of phopsho-TDP-43 mislocalization in a *C9ORF72* repeat expansion mouse model of ALS [56] (*C9ORF72* repeat expansion mutations are the most common cause of familial ALS). Importantly, impairment of SG assembly via Ataxin-2 depletion and chemical inhibitors of eIF2α phosphorylation can suppress nucleocytoplasmic trafficking defects and *C9ORF72* ALS models of neurodegeneration [55]. These data suggest that TDP-43 accumulation in the cytoplasm, possibly driven by SG assembly, impairs nucleocytoplasmic trafficking.

Given this, a notable result with our yeast TDP-43 induction model is that at early timepoints, where TDP-43 protein expression is likely still increasing, TDP-43 first accumulates diffusely and to a modest degree in the nucleus, shortly followed by formation of foci, approximately 20% of which (Figure 1A,C) appear to be perinuclear in nature (and independent of SGs). This is reminiscent of TDP-43 perinuclear foci observed in human cells [53] and hints at SG-independent and -dependent means of perturbing nucleocytoplasmic transport.

To assess the role of SG and PB assembly on TDP-43, we examined numerous yeast assembly mutants and SG/PB protein over-expression strains for effects on TDP-43 foci formation, toxicity and levels, in the hope that common patterns may emerge. While effects were observed, a key point is that no consistent trends emerge linking a specific alteration in SG or PB behavior to these TPD-43 phenotypes. Furthermore, TDP-43 toxicity in yeast cannot be predicted based on its propensity to form foci or overall TDP-43 abundance (Figure 2, Figure 3 and Figure S2–S4). Notably, *PUB1* (TIA-1 homolog), a key SG assembly factor in yeast [37,38], exhibited clearly reduced TDP-43 foci and a very slight decrease in toxicity when deleted (Figure 2), versus a clearer increase in toxicity and TDP-43 foci levels when over-expressed (Figure 3). Another interesting result was with *SCD6*, a translation repressor protein that localizes within and promotes P-body assembly in human cells [57,58] and also induces SGs in yeast when over-expressed [42,59]. *SCD6* over-expression revealed the most striking increase in TDP-43 foci levels and size and a clear enhancement of TDP-43 toxicity (Figure 3). Thus, a small subset of yeast SG/PB assembly promoting proteins seemingly facilitate TDP-43 aggregation and toxicity. Conversely, over-expression of eIF4G proteins, both of which are also implicated in SG assembly, like Pub1 [37], actually suppress TDP-43 toxicity. Additionally, *edc3Δ pat1Δ* cells, despite significantly limiting TDP-43 foci formation and overall abundance (Figure S2), have no impact on TDP-43 toxicity.

Numerous potential explanations exist for the lack of a clear correlation between SG and PB assembly and TDP-43 phenotypes in yeast. These include limiting experimental issues, such as variable effects on SG/PB assembly in our null and over-expression strains. Additionally, the extent of SG or PB assembly may matter less than the specific composition or dynamics of either SGs or PBs with regards to TDP-43 behavior. It is also possible that SG/PB-assembly-independent roles for gene deletions or over-expressions may explain the TDP-43 phenotypes we observe and that SG/PB assembly per se is not important. For example, while *BRE5* and *UPB3* (G3BP1 and USP10 homologs) promote SG assembly [41], they also form a ubiquitin protease complex that could in principle impact TDP-43 protein aggregation and stability. Indeed, TDP-43 levels are strongly reduced in *bre5Δ* and *ubp3Δ* yeast strains [43]. Carefully controlled experiments to selectively regulate SG and PB assembly while assessing composition and dynamics will be required to fully address this issue. Nonetheless, a closer analysis of the role of PBs in regulating TDP-43 may be warranted in human cells, given our findings with *SCD6* over-expression (Figure 3), *edc3Δ pat1Δ* P-body assembly mutants (Figure S2), and the progressive localization of TDP-43 to PBs in yeast over an acute stress time course (Figure S1).

Our data suggest that SG assembly in human cells may modestly contribute to maintenance of TDP-43 protein levels, specifically during recovery from acute arsenite stress (Figure 6A,B). This appears due to TDP-43 being more susceptible to proteasomal turnover in *G3BP1/2ΔΔ* mutants, given that TDP-43 levels are restored to unstressed levels in the presence of the proteasomal inhibitor MG132 (Figure 6C,D). TDP-43 turnover, in a myriad of contexts (WT, mutant, distinct cell types, stresses, aging, etc.), has been argued to occur by various mechanisms, including proteasomal, autophagic, chaperone-mediated autophagic and endocytic-based mechanisms [29,60,61,62,63,64], though no study to our knowledge has examined TDP-43 turnover in a context blocked for SG assembly. A simple model is that cytoplasmic TDP-43 remains more soluble and accessible to proteasomal turnover when unable to localize within and form larger assemblies/aggregates within SGs. An alternative possibility is that absence of G3BP1 and 2 directly alters TDP-43 levels in an SG-independent manner. Indeed, G3BP1 was recently described to promote proteasomal function and inhibit accumulation of ubiquitinated protein aggregates associated with Parkinson’s (α-synuclein) and Cystic Fibrosis (CFTR-ΔF508), which were induced by p62 and USP10. Interestingly, G3BP2 acted as an antagonist to these G3BP1 functions, with *G3BP1/2ΔΔ* mutants exhibiting intermediate phenotypes [65]. If extrapolated directly to TDP-43, we would expect an increase in TDP-43 protein abundance in *G3BP1/2ΔΔ* cells, contrary to our observations, though this work was not conducted in the context of stress or stress recovery. To further complicate matters, USP10 impairs SG assembly [32], whereas p62 has been implicated in SG clearance [19]. Clearly, work remains to be done here to carefully untangle SG assembly effects from G3BP1/2-specific effects, which represents an area of future interest.

While TDP-43, by virtue of its IDR and RNA-binding activity, likely can accumulate in numerous RNP compartments rich in IDR-containing proteins (e.g., SGs, PBs, nuclear pores), a key issue is the extent to which TDP-43 interactions, abundance, conformation and modification status drive distinct TDP-43 localization and aggregation behaviors. Recent work demonstrated that TDP-43 harbors poly(ADP-ribose) (PAR)-binding activity in domains that overlap its NLS, and this binding activity is critical to TDP-43 localization in mammalian cell and neuronal SGs [45], which are enriched in this modification [66]. While evidence supporting a PAR-dependent SG localization mechanism was clear, it is noteworthy that TDP-43 also localizes in *Saccharomyces cerevisiae* SGs, despite the absence of this modification and PAR-polymerases (PARPs).

## 5. Conclusions

In summary, our work adds to the emerging idea that SGs (and maybe PBs) can facilitate TDP-43 cytoplasmic mislocalization and aggregation but are not essential for the process. Supporting this, TDP-43 cytoplasmic foci first form in the majority of cases with SGs (Figure 1, Figure 4 and Figure 5)—see also [25]. Furthermore, a strong block (*G3BP1/2ΔΔ*) to SG assembly in mammalian cells completely impairs the formation of cytoplasmic TDP-43 foci under acute high arsenite stress (Figure 5) and reduces formation of TDP-43 cytoplasmic foci during and following a low arsenite chronic stress (Figure 5). Notably though, visible SG foci very rarely appear under such chronic stress conditions in WT cells, and localization with TDP-43 foci is also rarely observed. This mimics observations in another study [45] and suggests either an extremely transient role for SGs in promoting TDP-43 foci formation, a role for sub-microscopic SGs in TDP-43 foci formation (which recent proteomic studies suggest may exist [67,68]) or other possibilities including indirect effects or G3BP1/2-specific effects. Select yeast SG- and PB-altered strains also exhibit effects on TDP-43 foci formation (Figure 2, Figure 3 and Figure S2), though there is no consistency in this dataset that points to a clear granule assembly-dependent trend. Besides this yeast data, evidence that TDP-43 cytoplasmic foci can form independently of SGs includes the fact that TDP-43 cytoplasmic foci still appear under chronic stress in *G3BP1/2ΔΔ* cells, albeit at a lower frequency (Figure 5B). Taken together, and in the context of recent literature findings discussed above, we suggest that SG assembly can facilitate TDP-43 cytoplasmic localization and aggregation within cytoplasmic foci but are likely not essential to the process.

## Supplementary Materials

The following are available online at https://www.mdpi.com/2218-273X/10/10/1367/s1, Figure S1: TDP-43 foci increasingly colocalize with PBs following induction, Figure S2: edc3Δ pat1Δ PB assembly mutants exhibit reduction of TDP-43 aggregation and protein level, Figure S3: Over-expression of PB proteins has variable effects on TDP-43 toxicity, Figure S4: GAL1-Over-expression of DED1 increases TDP-43 foci formation without affecting TDP-43 toxicity, Figure S5: Validation of TDP-43 antibody specificity, Table S1: Strains, Plasmids and Antibodies used in this study, Table S2: Microscopy quantitation.
