# Correction: Fernandes et al. Stress Granule Assembly Can Facilitate but Is Not Required for TDP-43 Cytoplasmic Aggregation. *Biomolecules* 2020, *10*, 1367

**DOI:** 10.3390/biom11121895

**Published:** 2021-12-17

**Authors:** Nikita Fernandes, Luke Nero, Shawn M. Lyons, Pavel Ivanov, Telsa M. Mittelmeier, Timothy A. Bolger, J. Ross Buchan

**Affiliations:** 1 Department of Molecular and Cellular Biology, University of Arizona, Tucson, AZ 85721, USA; nikferns7@email.arizona.edu (N.F.); lnero@email.arizona.edu (L.N.); telsa@arizona.edu (T.M.M.); tbolger@email.arizona.edu (T.A.B.); 2 Department of Medicine, Harvard Medical School, Boston, MA 02115, USA; smlyons1@bu.edu (S.M.L.); pivanov@rics.bwh.harvard.edu (P.I.); 3 Division of Rheumatology, Immunity and Inflammation, Brigham and Women’s Hospital, Boston, MA 02115, USA

In the original article [1], there was a mistake in Figure 1 as published. The Pab1-GFP and TDP-43-mRuby labels in 1A accidentally got switched in final proofing. The corrected Figure 1 appears below.

There was an error in the original article. The text in the Abstract and at the end of the Introduction concerning phospho-TDP-43 localization in nucleoli in SG assembly mutants was accidentally left in place from a prior version of the paper. Specifically, the following sentences were removed: “Interestingly, in SG assembly mutant cells (*G3BP1/2*ΔΔ), TDP-43 is enriched in nucleoli” (Abstract). “Additionally, cells deficient in SG assembly (*G3BP1/2*ΔΔ), exhibit a striking accumulation of phospho TDP-43 in nucleoli” (End of introduction).

This error stemmed from initial data with an antibody whose reliability came into question given control experiments with TDP-43 KO cell lines, which yielded similar results as in WT cells. This issue was identified prior to final peer review, and data associated with said antibody were removed from the Results and Discussion sections (but not the Abstract or Introduction); appropriate discussion of this finding was included in the Results (Section 3.5).

The authors apologize for any inconvenience caused and state that the scientific conclusions are unaffected. The original article has been updated.

## Figures and Tables

**Figure 1:**
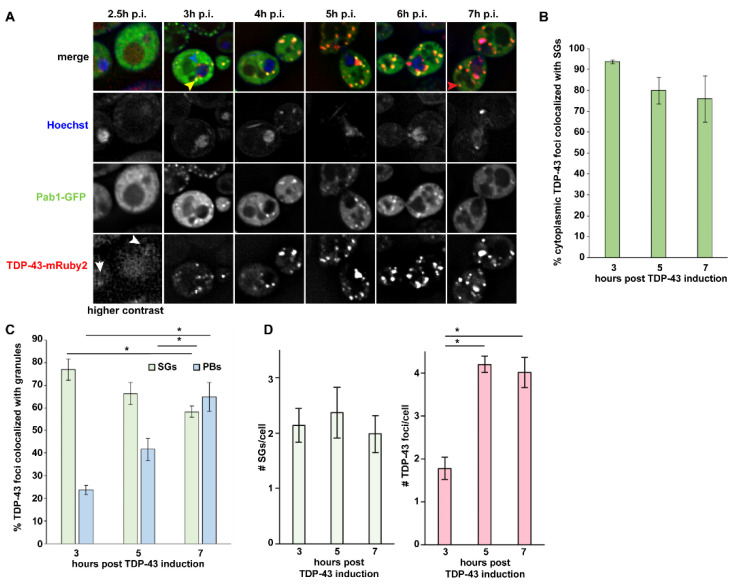
TAR DNA-binding protein 43 (TDP-43) foci predominantly initiate with stress granules (SGs). (**A**) Log-phase Saccharomyces cerevisiae BY4741 (WT) were transformed with pRB194-expressing TDP-43-mRuby2 and pRB015 expressing Pab1-GFP (SG marker). Hoescht was used to visualize the nucleus. TDP-43 expression induced by 2% Galactose, with timepoints post induction (p.i.) microscopically imaged as indicated. Arrowheads indicate phenotypes mentioned in the text. (**B**) % colocalization of cytoplasmic TDP-43 foci with Pab1-GFP (perinuclear-associated foci excluded). Data generated from 3 biological replicates with mean ± s.d shown. (**C**) % colocalization of all TDP-43 foci with Pab1-GFP (including peri-nuclear TDP-43 foci) or Dcp2-GFP (see Figure S1). Data generated from 3 biological replicates with mean ± s.d shown. An ANOVA with Tukey’s post-hoc test was used to assess significance (* indicates significance). (**D**) Total SG and TDP-43 cytoplasmic foci number; replicates and error bars as in (**C**). An ANOVA with Tukey’s post-hoc test was used to assess significance.
