# Supplementary material for: Stress Granule Assembly Can Facilitate but Is Not Required for TDP-43 Cytoplasmic Aggregation
Source: Biomolecules. 2020 Sep 25;10(10):1367. doi: 10.3390/biom10101367 (PMC7650667; doi:10.3390/biom10101367)
Supplement: Supplementary file 1 [file biomolecules-10-01367-s001.zip › biomolecules-942451-supplementary/Suppl Figure Legends.docx]

Supplementary Figure Legends

**Figure S1. TDP-43 foci increasingly colocalize with PBs following induction.** (**A**) As in Figure 1A except cells transformed with pRB038 expressing Dcp2-GFP (PB marker). Yellow arrowhead indicates co-localization. (**B**) Quantitation of (**A**).

**Figure S2. edc3Δ pat1Δ PB assembly mutants exhibit reduction of TDP-43 aggregation and protein level.** (**A**) Log-phase wild-type S. cerevisiae RP840 (WT), edc3Δ, lsm4ΔC, edc3Δlsm4 ΔC, pat1Δ, and edc3Δpat1Δ strains were transformed with pRB194 expressing TDP-43-mRuby2 and examined for the presence of foci. (**B**) Quantification of B; % cells with TDP-43 foci. Data generated from three biological replicates with mean ± s.d shown. ANOVA with Dunnett’s post-hoc test was used to assess significance. (**C**) Western analysis of above strains to assess total TDP-43-mRuby2 protein levels, normalized to the GAPDH loading control.

**Figure S3: Over-expression of PB proteins has variable effects on TDP-43 toxicity.** (**A**) PB assembly protein over-expression strains; data generated as in Figure 3A. (**B**) BRE5 and UBP3 are deubiquitinases implicated in SG assembly. Data generated as in Figure 3A.

**Figure S4: GAL1-Over-expression of DED1 increases TDP-43 foci formation without affecting TDP-43 toxicity.** (**A**) Log-phase W303 (WT) was transformed with pRB427 expressing GAL1-DED1 or the pRB428 empty vector and pRB112 expressing TDP-43-GFP. Cells were examined for TDP-43 foci formation by microscopy. (**B**) The above strains (rows 2 and 4) along with strains containing the pRB428 empty vector and GAL1-DED1 (row 3) or pRB384 (second empty vector; row 1) were examined for growth by serial dilution spotting assay.

**Figure S5: Validation of TDP-43 antibody specificity.** (**A**) HEK293 cells expressing endogenous TDP-43 and lentiviral-integrated WT TDP-43-GFP or TDP-35-GFP were assessed for TDP-43 levels using Abcam ab290 (“GFP Ab”) and other TDP-43 antibodies as indicated. Arrows indicate various GFP-labelled and non-labelled TDP-43 bands and breakdown products. (**B**) U2-OS (WT), G3BP1/2ΔΔ, and TDP-43Δ cells were assessed for endogenous TDP-43 levels with indicated antibodies. C) U2-OS (WT) and TDP-43Δ cells were assessed for localization of TDP-43 by immunofluorescence using the same TDP-43 Abs as in A.
