# Supplementary figures and images for: Stress Granule Assembly Can Facilitate but Is Not Required for TDP-43 Cytoplasmic Aggregation
Source: Biomolecules. 2020 Sep 25;10(10):1367. doi: 10.3390/biom10101367 (PMC7650667; doi:10.3390/biom10101367)

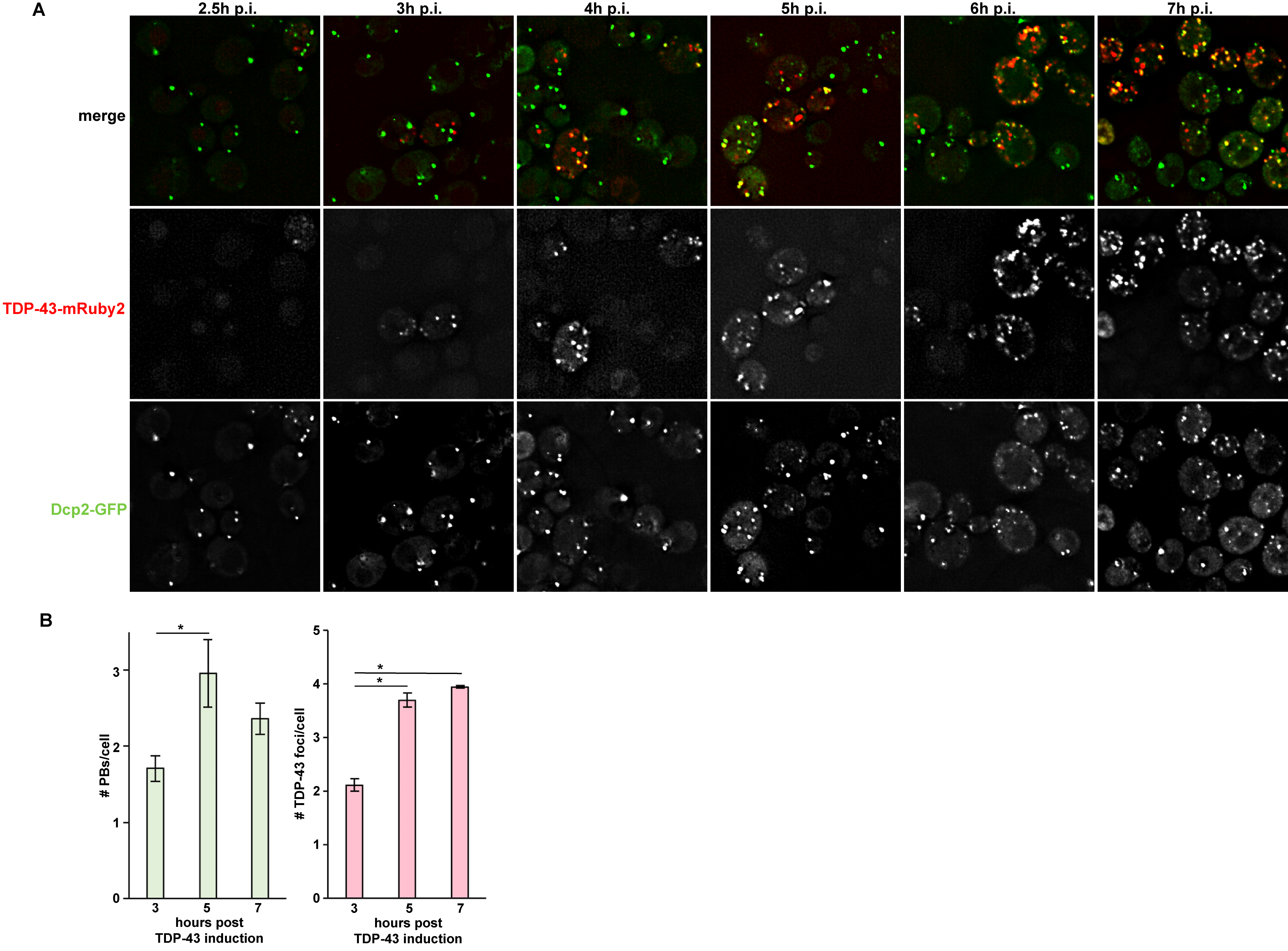

Supplement: Supplementary file 1 [file biomolecules-10-01367-s001.zip › biomolecules-942451-supplementary/FigS1.tif]

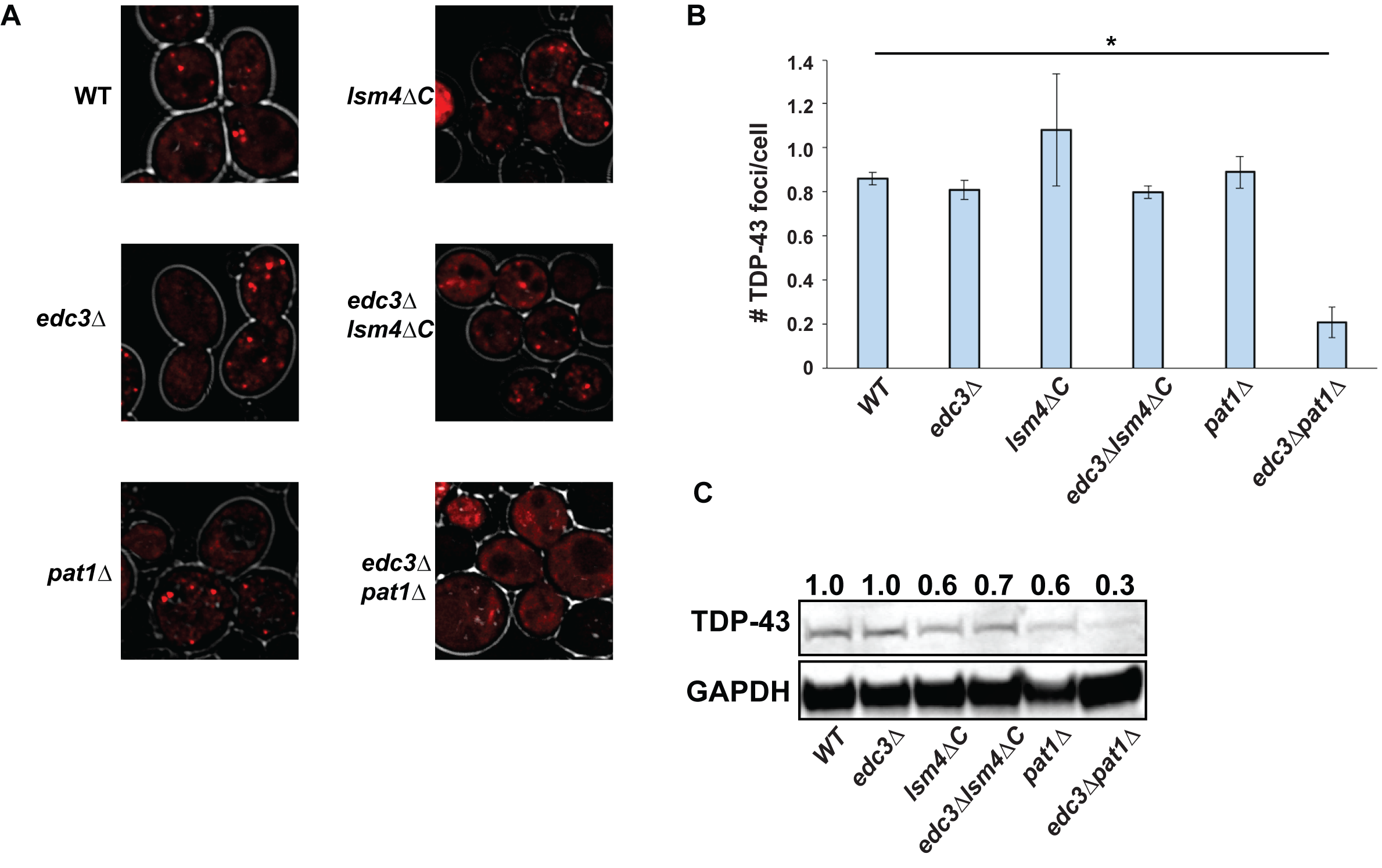

Supplement: Supplementary file 1 [file biomolecules-10-01367-s001.zip › biomolecules-942451-supplementary/FigS2.tif]

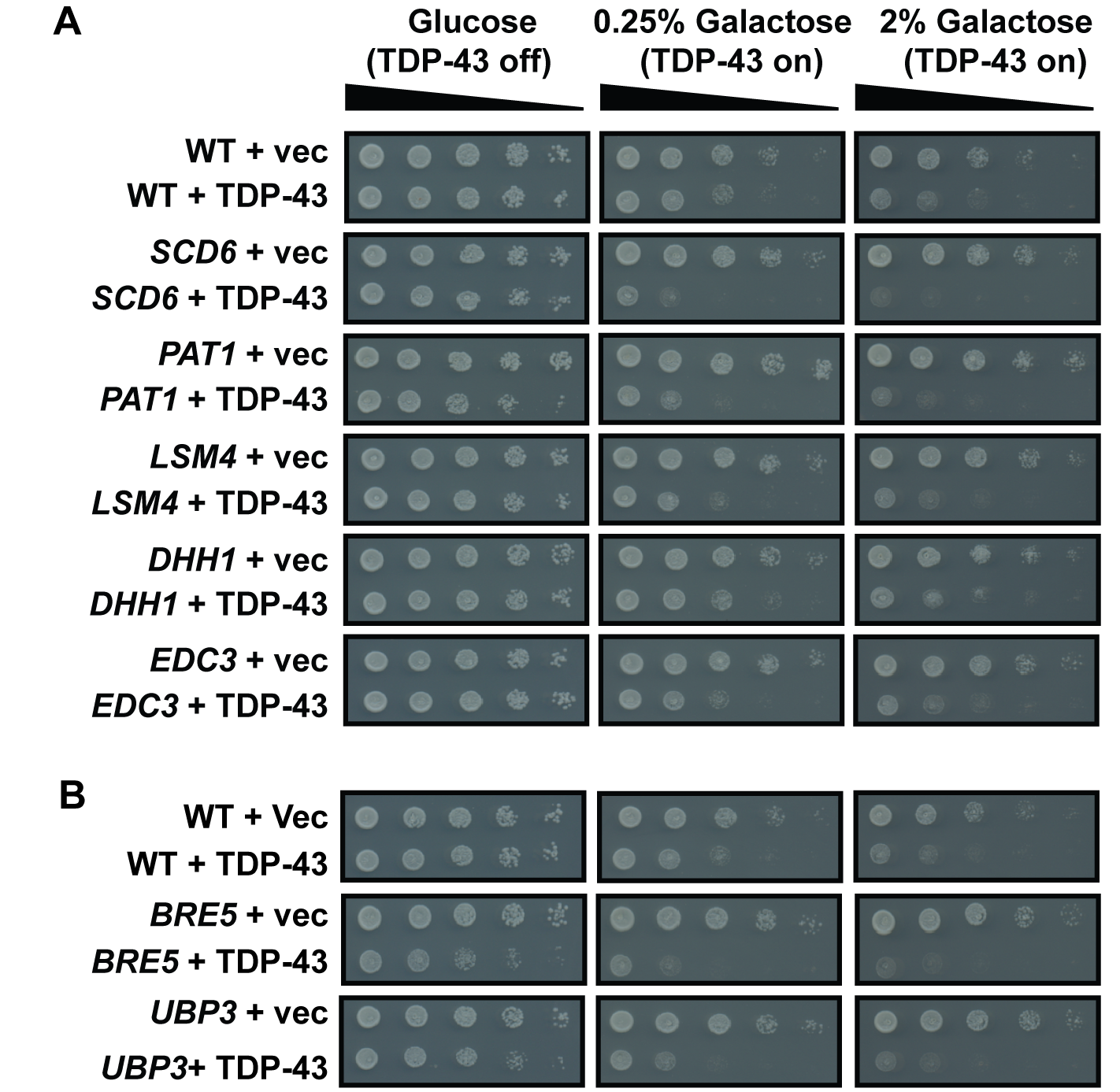

Supplement: Supplementary file 1 [file biomolecules-10-01367-s001.zip › biomolecules-942451-supplementary/FigS3.tif]

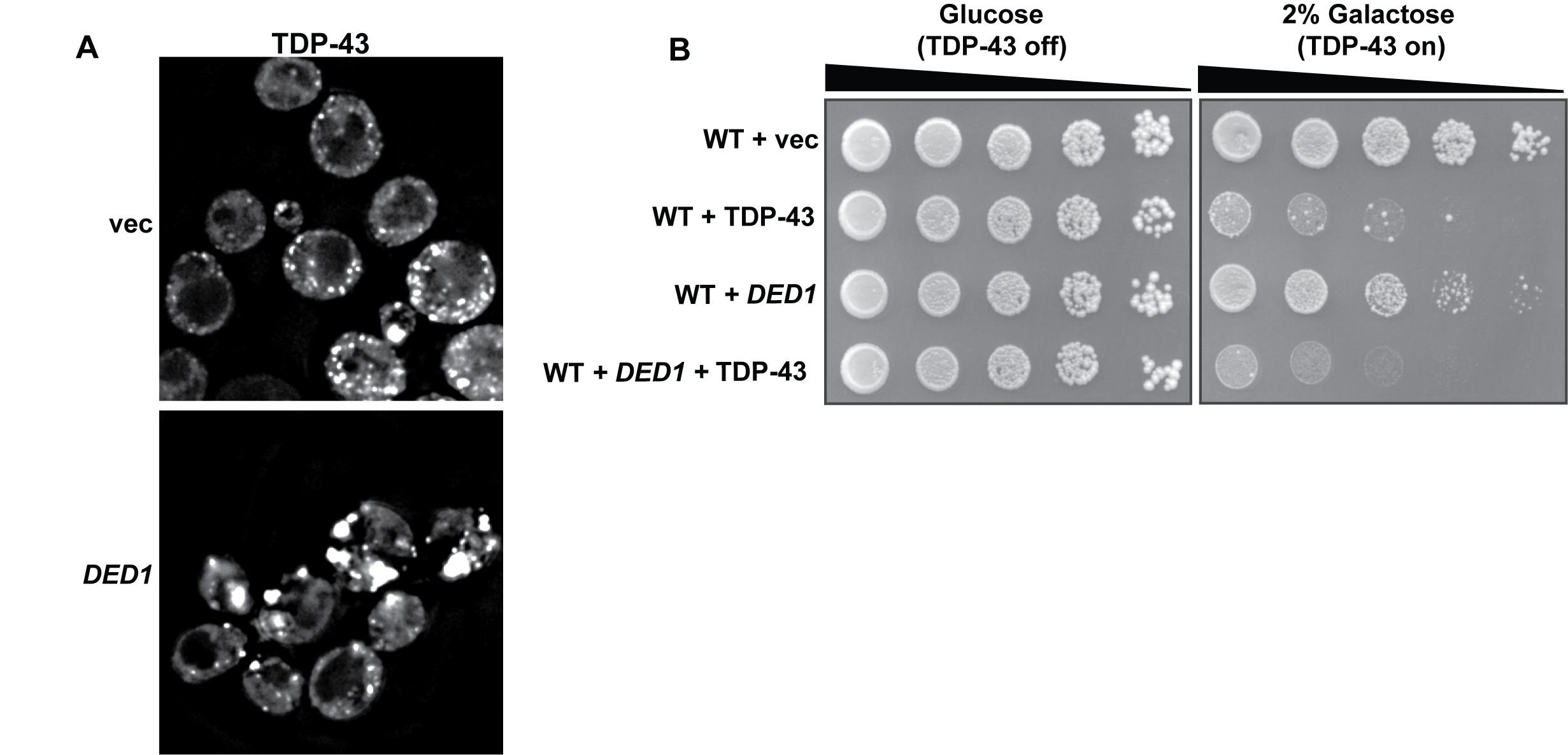

Supplement: Supplementary file 1 [file biomolecules-10-01367-s001.zip › biomolecules-942451-supplementary/FigS4.tif]

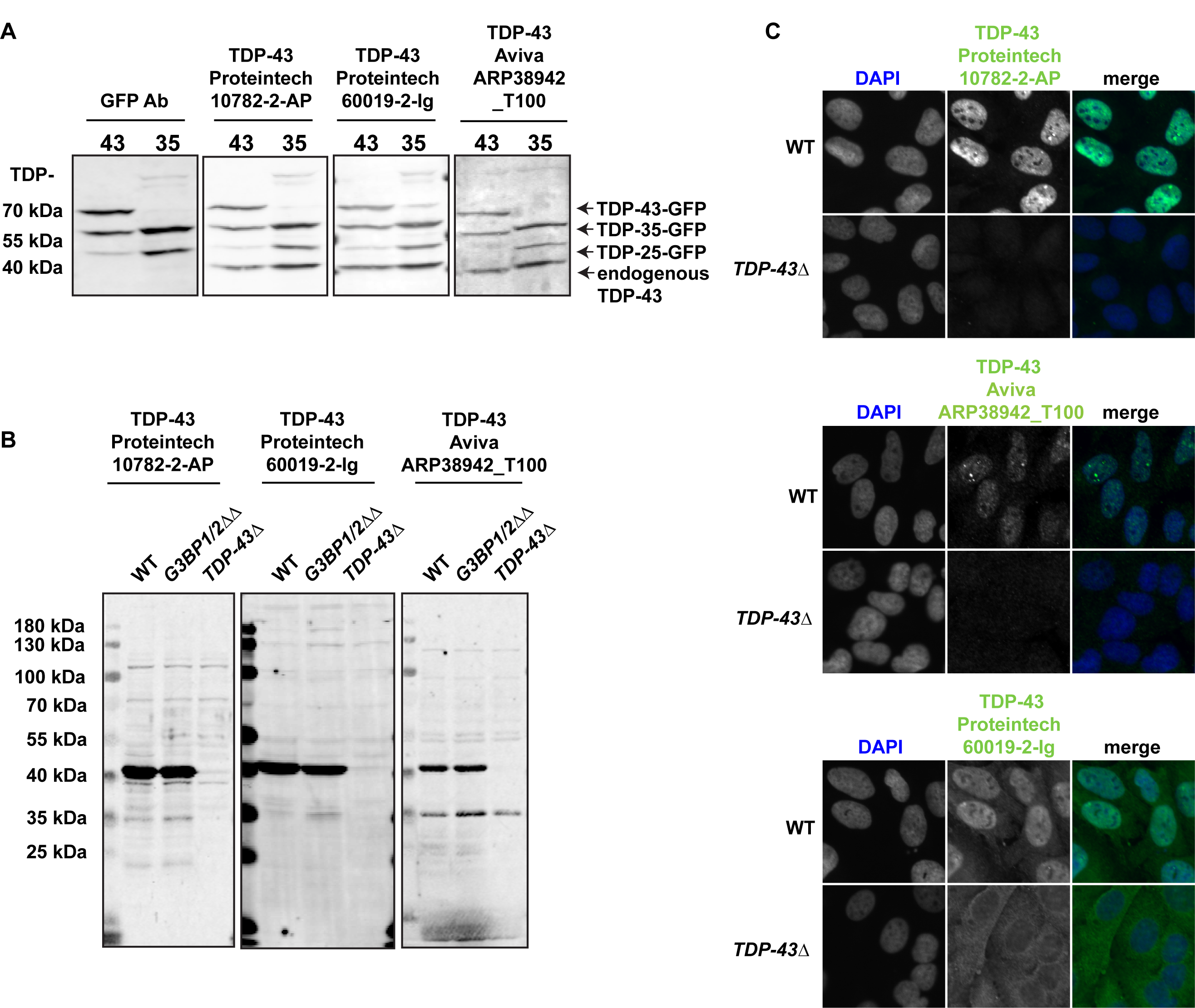

Supplement: Supplementary file 1 [file biomolecules-10-01367-s001.zip › biomolecules-942451-supplementary/FigS5.tif]
